# The success of behavioral economics in improving patient retention within an intensive primary care practice

**DOI:** 10.1186/s12875-021-01593-8

**Published:** 2021-12-22

**Authors:** Phillip Groden, Alexandra Capellini, Erica Levine, Ania Wajnberg, Maria Duenas, Sire Sow, Bernard Ortega, Nia Medder, Sandeep Kishore

**Affiliations:** 1 Icahn School of Medicine at Mount Sinai, 1 Gustave L. Levey Place, Box 1199, New York, NY 10029 USA; 2 Arnhold Institute for Global Health, Icahn School of Medicine at Mount Sinai, 1216 5th Avenue, Box 1199, New York, NY 10029 USA; 3 Department of General Internal Medicine, Icahn School of Medicine at Mount Sinai, 1 Gustave L. Levy Place, Box 1087, New York, NY 10029 USA; 4 School of Medicine, University of California San Francisco, 533 Parnassus Ave, San Francisco, CA 94143 USA

**Keywords:** High-cost populations, Chronic care, Patient retention, Behavioral economics

## Abstract

**Background:**

A minority of the U.S. population comprises a majority of health care expenses. Health system interventions for high-cost populations aim to improve patient outcomes while reducing costly over-utilization. Missed and inconsistent appointments are associated with poor patient outcomes and increased health care utilization. PEAK Health— Mount Sinai’s intensive primary care clinic for high-cost patients— employed a novel behavioral economics-based intervention to reduce the rate of missed appointments at the practice. Behavioral economics has accomplished numerous successes across the health care field; the effect of a clinic-based behavioral economics intervention on reducing missed appointments has yet to be assessed.

**Methods:**

This was a single-arm, pre-post trial conducted over 1 year involving all active patients at PEAK Health. The intervention consisted of: a) clinic signage, and b) appointment reminder cards containing behavioral economics messaging designed to increase the likelihood patients would complete their subsequent visit; appointment cards (*t1*) were transitioned to an identical EMR template (*t2*) at 6 months to boost provider utilization. The primary objective, the success of scheduled appointments, was assessed with *visit adherence*: the proportion of successful over all scheduled appointments, excluding those cancelled or rescheduled. The secondary objective, the consistency of appointments, was assessed with a 2-month *visit constancy* rate: the percentage of patients with at least one successful visit every 2 months for 1 year. Both metrics were assessed via a χ^2^ analysis and together define *patient retention*.

**Results:**

The *visit adherence* rate increased from 74.7% at baseline to 76.5% (*p = .22*) during *t1* and 78.0% (*p = .03*) during *t2*. The 2-month *visit constancy* rate increased from 59.5% at baseline to 74.3% (*p = .01)* post-intervention.

**Conclusions:**

A low-resource, clinic-based behavioral economics intervention was capable of improving *patient retention* within a traditionally high-cost population. A renewed focus on *patient retention*— employing the metrics described here— could bolster chronic care efforts and significantly improve the outcomes of high-cost programs by reducing the deleterious effects of missed and inconsistent appointments.

## Background

Studies demonstrate that within the United States, a small percentage of the population comprises a significant portion of health care expenses. Analyses across multiple groups—including Veterans Affairs, Medicare, and Medicaid populations—estimate that 5% of patients account for nearly half of annual spending [1–4]. While not all of these patients are consistently high-cost, a cohort of individuals remains so over time [5]. Persistently high-cost patients are afflicted with numerous chronic conditions and psychosocial needs while lacking access to consistent care. These factors undermine their ability to manage their health and lead to an increase in preventable emergency department (ED) utilization and hospital admissions. Health systems have implemented a variety of programs designed for high-cost patients, ranging from care coordination efforts to specialized primary care practices [6–10]. The shared goals of these programs are two-fold: to improve patient outcomes while reducing health care utilization and spending within such populations.

Missed appointments are a ubiquitous issue for medical practices. Rates for adult primary care practices range from 15 to 33% [11]. In addition to decreasing clinic revenue and productivity, missed appointments adversely impact both health care utilization and patient outcomes [12]. Within academic primary care practices, patients with a propensity for missed appointments have significantly higher rates of ED visits and hospitalizations compared to all other individuals. These same patients exhibit worse clinical outcomes than those less likely to miss appointments, including lower cancer (colorectal, cervical, and breast) screening rates and above-goal low-density lipoprotein and hemoglobin A1c measures [13]. The consistency of appointments has also been shown to impact health care utilization, as ambulatory practice patients with a higher continuity of care demonstrate a decreased risk of ED visits [14].

Since the deleterious effects of missed and inconsistent appointments directly oppose the goals of high-cost programs, PEAK Health—Mount Sinai’s intensive primary care program for its high-cost population—sought to reduce the rate of missed visits within its practice. The group partnered with the behavioral economics firm VAL Health to implement a low-resource intervention capable of improving patients’ adherence to scheduled appointments. Behavioral economics combines the field of psychology with economic theory to employ the subconscious aspects of our decision-making processes; interventions based upon these tenets harness our unconscious biases to ‘nudge’ individuals towards a favorable outcome.

The ability of a clinic-based behavioral economics intervention to reduce a practice’s missed appointment rate, especially within a population traditionally disconnected from care, has yet to be determined. The primary objective of this study was to assess whether the intervention improved PEAK Health patients’ adherence to scheduled appointments. A secondary objective was to determine if the intervention simultaneously increased the consistency of patients’ appointments over time. Both of these outcomes—the success and consistency of patients’ engagement with their source of care—comprise *patient retention*. We hypothesized such a behavioral economics intervention could improve high-cost patients’ retention in care through increases in both *visit adherence* and *visit constancy*.

## Methods

### Study Design & Setting

This was a single-arm, pre-post interventional trial conducted at Mount Sinai’s PEAK Health practice over the course of a year between April 2018 to March 2019— prior to the emergence of COVID-19 in the United States. The study was determined to not meet the definitions of Humans Subject Research and was considered exempt from IRB review and oversight. The PEAK Health program is an intensive primary care clinic located in East Harlem that provides targeted care for the health system’s high-cost population. Patients are referred to the practice through numerous sources—including the ED, primary care practices, and via risk-based analytics tools—and are eligible to enroll if they exhibit high health care utilization (i.e. ED visits or inpatient admissions), uncontrolled chronic conditions, and psychosocial drivers of their barriers to and patterns of care.

Patients are assigned to multidisciplinary ‘pods’ consisting of a treating provider (MD or NP), a licensed social worker, and a care coordinator. Within the program’s transitional care model, patients’ health goals and barriers are identified and reached through successive multidisciplinary visits before ‘graduating’ individuals back to a traditional primary care provider. The clinic employs extended appointments, frequent follow-up, the capacity for urgent and home visits, and an emphasis on holistic, preventative health. At the close of the study period, PEAK Health had 286 patients enrolled in its program. All patients active at the practice during the study period were privy to the components of the intervention.

### Intervention

**Fig. 1:**
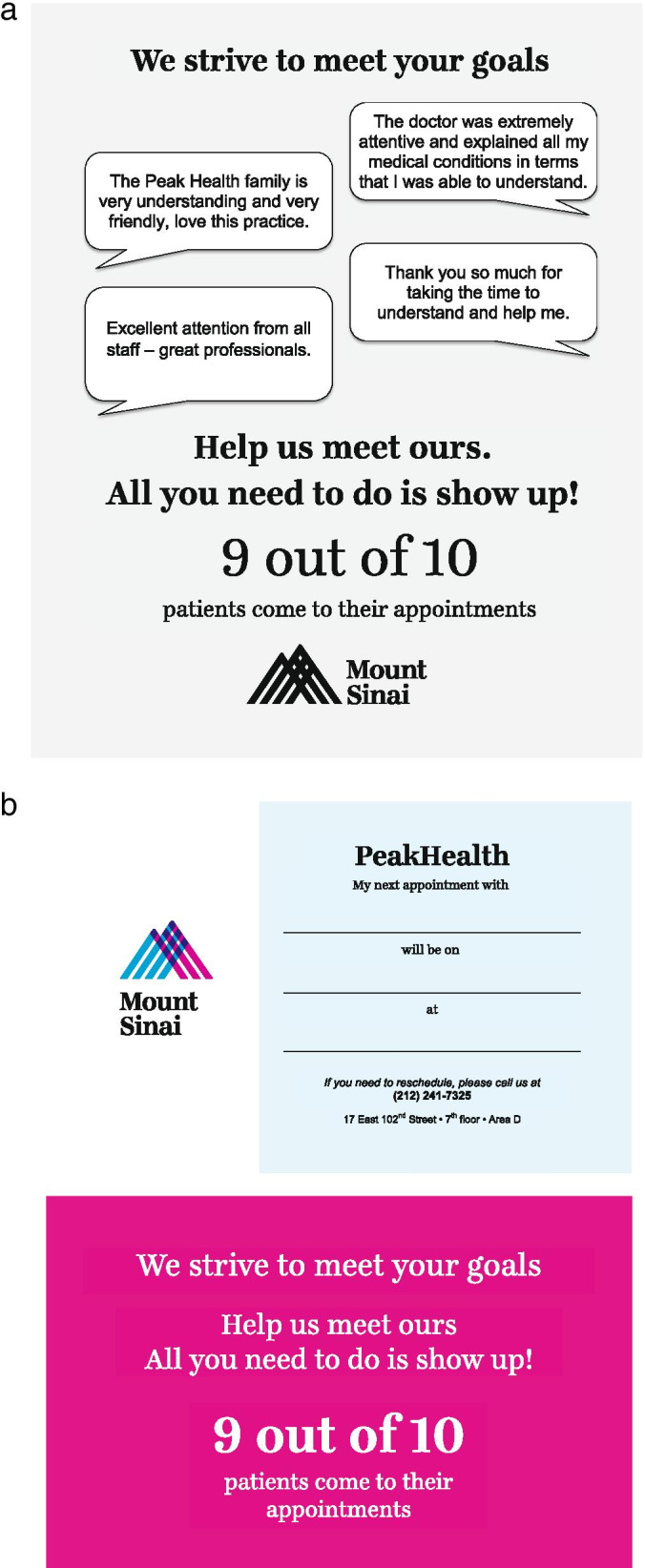
**a** Template of the clinic signage component of the behavioral economics intervention, featured within PEAK Health’s waiting and exam rooms. **b** Template of the appointment reminder card component of the behavioral economics intervention, located next to each provider workstation. The format and language were mirrored within an EMR template that was introduced at 6-months post-implementation to boost adoption

The study intervention consisted of specialized: 1) clinic signage featured throughout the practice (Fig. 1a), and 2) appointment reminder cards located at provider workstations (Fig. 1b). Both components were developed by ValHealth using proven behavioral economics concepts (e.g. social norms, saliency, pledging) that were designed to increase the likelihood patients would return for their subsequent appointment. The signage was located at the front of the clinic’s waiting area and within each exam room, such that patients engaged with the material at each stage of their visit. Appointment reminder cards— located in each exam room— were intended to be completed and signed by patients at the close of each visit to confirm the date and time of their next appointment.

Both components were implemented at PEAK Health simultaneously and remained in the clinic for the 12-month study period. Adoption of the intervention was ascertained through frequent check-ins with clinic staff. Midway through the study, the appointment reminder card was transitioned to an electronic medical record (EMR) template due to decreased utilization of the former by clinic staff; the EMR template mirrored the language of the physical card and was implemented to boost adoption via integration of this component into staff’s existing workflow. Providers included the templates within a printed ‘After-Visit Summary’ and, similar to the appointment reminder cards, instructed patients to complete and sign them at the close of each visit. Successful adoption of the template by clinic providers was confirmed by reviewing ‘After-Visit Summary’ within the EMR following implementation of this component at 6 months.

### Outcome metrics & statistical analysis

The primary objective, the success of scheduled appointments, was assessed by calculating the practice’s *visit adherence* rate: the proportion of completed appointments over all scheduled appointments, excluding those that were cancelled or rescheduled. *Visit adherence* rates were calculated from appointment outcome data tracked by administrative staff and compared between the study’s baseline (6-months pre-implementation) and intervention (12-months post-implementation) periods via a χ^2^ analysis. The intervention period was divided into two 6-month blocks representing implementation of the physical appointment reminder card (*t1*) versus the EMR template (*t2*).

The secondary objective, the consistency of appointments over time, was assessed by calculating each patient’s *visit constancy* rate, defined as a proportion of time intervals containing at least one completed appointment over a single year. Follow-up appointments at PEAK Health are scheduled every 2 weeks to 2 months depending on patients’ care needs; the *visit constancy* time interval was thus set at 2-months to capture this entire range of appointments. Lists of patients were generated pre- and post-intervention, and the number of 2-month intervals with at least one completed appointment was assessed for each patient over the previous year. Completed appointments were confirmed via the presence of PEAK Health’s provider notes within the EMR, and intervals in which patients were intentionally not scheduled were excluded from the analysis. The percentage of patients that achieved a perfect (100%) *visit constancy* rate (i.e. completed an appointment within each 2-month interval annually) was compared pre- and post-intervention by a χ^2^ analysis.

## Results

Demographic information for the PEAK Health program at the start of the intervention period has been provided (Table 1). Briefly, 58.2% of the patients identify as female, 34.2% of patients identified their race as African American or Black, and the average age of the population is 59 years old (range: 20 to 100 years old).

**Table 1:** Demographic information for Mount Sinai’s PEAK Health clinic

Sex	
Female	58.2%
Race (Self-Reported)	
African American or Black	34.2%
White	5.0%
Other^a^	60.8%
Age	
0–20	0.3%
21–40	12.9%
41–60	38.6%
61–80	40.7%
81–100	7.4%

^a^ 79% of “Other” self-identified their ethnicity as Hispanic or Latino

For the primary objective, 1716 expected appointments were scheduled at PEAK Health during the 6-month baseline period (2320 appointments booked; 604 cancelled or rescheduled). Within the intervention period, 3218 expected appointments were scheduled (4348 appointments booked; 1130 cancelled or rescheduled); 1701 of these appointments were scheduled during *t1* (558 appointments cancelled or rescheduled), and 1517 were scheduled during *t2* (572 appointments cancelled or rescheduled). PEAK Health patients’ missed appointments decreased from a total of 434 in the baseline period compared to 399 during *t1* and 333 in *t2*. Notably, the practice’s *visit adherence* rate increased from 74.7% (1282/1716) at baseline to 76.5% (1302/1701; *p* = .21, *95% CI*: − 1.0 to 4.7%) during *t1* and 78.0% (1184/1517; *p =* .03, *95% CI*: 0.4 to 6.3%) during *t2*— the latter representing a significant increase in the *visit adherence* rate compared to baseline. The rate of cancelled or rescheduled appointments did not differ throughout the study. 26.0% (604/2320) of all appointments were cancelled or rescheduled during the baseline period compared to 24.7% (558/2259; *p* = .31, *95% CI*: − 1.2 to 3.6%) during *t1* and 27.3% (572/2089; *p* = .31, *95% CI*: − 1.3 to 4.0%) during *t2*.

For the secondary objective, a total of 84 patients were active during all 2-month intervals at the close of the baseline period, the year prior to implementation of the intervention; at the close of the study period, 1 year post-implementation, 199 patients were active during all 2-month intervals. In the baseline period, 59.5% of patients (50/84) achieved a perfect 2-month *visit constancy* rate, or completed at least one visit in every 2-month interval over the year; following implementation of the intervention, the number of patients that achieved a perfect 2-month *visit constancy* rate increased to 74.3% (148/199; *p* = .01, *95% CI*: 3.1 to 26.9%), representing a significant increase in the number of patients attaining consistent care at PEAK Health after deploying the behavioral economics intervention.

## Discussion

The objective of this study was to determine if a clinic-based, behavioral economics intervention could improve the success and consistency of appointments within an intensive primary care program for high-cost patients. Behavioral economics has accomplished a variety of successes in the health care field, from increasing cancer-screening rates to altering prescriber ordering practices [15, 16]. One study in particular proved that behavioral economics-framed text message (SMS) reminders were successful in similarly reducing a health system’s missed appointment rate [17]. While other interventions have utilized SMS-based reminders to increase appointment adherence across a variety of settings, this study represents the only other application of a behavioral economics framework to promote a facet of *patient retention* [18–20].

Here, we have uniquely proven that a behavioral economics intervention is capable of improving both patients’ adherence to scheduled appointments *and* their consistency of appointments across time— the latter finding being of particular relevance to chronic care providers. Furthermore, in contrast to the SMS-based systems, these improvements have been accomplished through a low-resource and clinic-based solution. Such an intervention could be deployed in any setting and might be coupled to an SMS-reminder system for increased benefit. Finally, these modest yet significant gains have been accomplished in a population of traditionally disconnected patients with a now increased appointment burden at little to no cost, further supporting the utility of a behavioral economics approach to *patient retention*.

The increase in *visit adherence* between *t1* (76.5%) and *t2* (78.0%) is likely attributable to increased adoption of the EMR template versus the physical appointment reminder card by the staff— proving that imbedding change within existing workflows is vital for implementation success. Furthermore, the stable rate of cancelled or rescheduled appointments between the baseline and study periods indicate the improvements in *visit adherence* were due to a true decrease in missed appointments opposed to an increase in simply calling ahead before not showing. Finally, the sizeable increase in *visit constancy* between the baseline (59.5%) and study periods (74.3%) amidst modest improvements in *visit adherence* suggests a group of imperfectly retained patients may have been more amenable to the affects of the intervention than others.

Key factors had the potential to confound the study’s outcomes. Adverse weather has a negative impact on *visit adherence*, and poor weather in the baseline period may have conflated the effects of our intervention [21]. Though not completely explanatory, the annual precipitation during the study was increased compared to baseline, suggesting adverse weather was not overly impactful [22]. Additionally, due to an unrelated effort by the clinic, the pool of active patients increased between the baseline and study periods (84 vs. 199); this latter cohort may have been more adherent to scheduled appointments independent of our intervention. However, PEAK Health’s census is inherently fluid due to the clinic’s approach to ‘graduating’ patients, and an increase in the number of new and historically disconnected patients might be theoretically less engaged with the clinic—suggesting this factor had minimal impact on our results.

Limitations of the study exist. Due to the data collection timeline, the baseline period for the primary objective (*visit adherence*) is 6-months shorter than the secondary objective (*visit constancy*), despite identical study period lengths. Division of the intervention period into two 6-month blocks (*t1* & *t2*) did allow for an equivalent analysis of the primary objective data, and identical yearlong periods were required for analysis of the secondary objective— as *visit constancy* is a proportion of successful appointments across time. Additionally, our patient visit data was not encoded by demographic parameters. While an analysis of the intervention’s efficacy by demographic subgroups, such as age or primary language, would have been useful to describe, these results were unfortunately not able to obtained.

Future studies ought to further determine the effects of enhanced *patient retention* on both practice and patient-specific outcomes metrics. This is especially relevant to programs for high-cost patients, where success is tied to improvements in these areas; within PEAK Health, patient cohorts exhibiting varying degrees of retention could be compared on attainment of health goals (e.g. hemoglobin A1c), length of time before program ‘graduation’, or ED utilization. Given the rise of telemedicine due to the COVID-19 pandemic, other studies might assess the feasibility of a behavioral economics intervention to boost *patient retention* within the virtual environment. As already mentioned, assessing the efficacy of our intervention within demographic groups or in combination with an SMS-based approach would also be worthwhile.

Despite the aforementioned benefits of retaining patients in care, standardized measures of *patient retention* are non-existent in the chronic care space. Both metrics presented are necessary to characterize *patient retention*, as each has its own benefits and restrictions. *Visit adherence* is limited in assessing retention in those lost to follow-up; such patients may have a high proportion of completed appointments while remaining disconnected from care overall. *Visit constancy* adds context by providing a view of successful appointments across time, though it fails to reflect retention in those requiring more frequent engagement. Such individuals may attend visits at regular intervals compared to average patients while still missing numerous appointments—a situation accurately captured by *visit adherence*. Ideal retention is therefore achieved through optimization of both metrics.

The value of *patient retention* as a component of high-quality care was originally demonstrated in the HIV care setting, where suboptimal retention was linked with substandard outcomes for such patients— leading to the publication of the metrics presented here in the literature [23, 24]. Just as the original authors posited that these measures “could have more widespread application” in “long-term disease management more broadly”, we similarly propose the employment of valid *patient retention* measures in the chronic care field [25]. Standardized metrics provide an objective method to evaluate *patient retention* and assess efforts, such as this study, to improve retention in care. Consistent tracking of retention data could inform real-time practice decisions, allowing providers to identify patients at-risk of becoming disconnected and intervene appropriately. Given the impact of adherent patients on staff productivity, clinic revenue, and continuity of care, all practices could benefit from improved *patient retention* [12].

As previously discussed, the deleterious effects of missed and inconsistent appointments directly oppose the two-fold goals of programs for high-cost populations: to improve health outcomes while reducing health care utilization and spending [13, 14]. Despite the theoretical benefit of high-cost programs, a number of initiatives have fallen short of these aims [9, 10, 26]. Inadequate *patient retention* is a plausible barrier for high-cost groups to attain these goals. If high-cost programs are a vehicle for the treatment of high-cost populations, *patient retention* is adherence to this treatment. Optimizing treatment adherence through successful and consistent appointments could be the key to achieving high-cost programs’ target goals— just as attaining blood pressure control is often achieved by simply increasing adherence to antihypertensive medications [27]. Indeed, certain programs recognize this and have begun publishing strategies for boosting engagement within their high-cost settings [28, 29]. Authors reporting on high-cost programs should consider publishing *patient retention* data as a means of contextualizing their outcomes; our readers are encouraged to utilize the metrics presented here to assist them.

## Conclusion

Considering just 5% of the population accounts for nearly half of all health care expenses in the United States, the success of programs for our high-cost population is vital for the sustainability of our nation’s health care system. Bolstering *patient retention* has the potential to significantly improve the outcomes of such programs through a reduction in the deleterious effects of missed appointments and inconsistent engagement. Behavioral economic strategies, such as the clinic-based intervention described here, have proven to be an effective, low-cost method for improving *patient retention*— even within a population of individuals traditionally disconnected from care. Employment of the metrics presented here provides a succinct method for our readers to evaluate *patient retention* within the chronic care space.

## Data Availability

The datasets generated and analyzed during this study are not publicly available due to clinic and patient privacy concerns but are available from the corresponding author on reasonable request.

## Abbreviations

ED: Emergency Department
EMR: Electronic Medical Record
SMS: ‘Text Message’ (Short Message Service)
